# Assessment of obesity management in medical examination

**DOI:** 10.1186/1475-2891-4-10

**Published:** 2005-03-03

**Authors:** Leo Treyzon

**Affiliations:** 1 Divisions of Digestive Diseases and Clinical Nutrition, Center for Human Nutrition, David Geffen School of Medicine at UCLA, 900 Veteran Avenue, Room 12–217, Los Angeles, CA 90095, USA

## Abstract

Obesity is a growing international health problem that has already reached epidemic proportions, particularly within the United States where a majority of the population is overweight or obese. Effective methods of treatment are needed, and should be taught to physicians by efficient means. There exists a disconnect between the rising obesity prevalence with its high toll on medical resources, and the lack of obesity education provided to practitioners in the course of their training. One particular shortfall is the lack of representation of obesity on standardized medical examinations. Physician attitudes toward obesity are influenced by their lack of familiarity with the management of the disease. This may include dietary restriction, increasing physical activity, behavior modification, pharmacotherapy, and surgical interventions. Thus, curricular changes in the medical education of obesity could help reduce morbidity and mortality associated with this disease.

## 

Wegeners's granulomatosis, errlichiosis, and tetrology of Fallot are examples of some of the rare diseases that I have learned to identify and look for in patients for over seven years as a medical student and resident in Internal Medicine. As a physician in training, I was frequently challenged to consider broad differentials and to watch out for the zebra that masquerades as a common disease throughout my clinical encounters. I have had my antennas up in search of these diseases for quite a while now. I have not, however, encountered any patients with these diseases so far. What I have usually encountered instead are common diseases, like type 2 diabetes, hypertension, and arthritis. More than anything, I have been struck by the incidence of obesity in our population. I find it alarming that many clinicians fail to actively address this growing epidemic in a formal manner with their patients, but rather regard obesity as a lifestyle choice that is the patient's sole responsibility.

My personal experience with obesity, having trained at a quaternary referral center in the relatively thin state of California, is that over one third of the patients I cared for were obese (not just overweight). Consistent with national trends, patients in my region have been becoming more obese and accordingly have been suffering increasingly from the complications of the disease, such as hypertension, insulin resistance, fatty liver disease, dyslipidemia, coronary heart disease, osteoarthritis, obesity-related cancer, and premature death [1]. I have been reassured that national policy makers at agencies such as the NIH and Medicare have taken administrative steps to address this epidemic [2]. What has perplexed me is that medical educators have been slow to prioritize obesity as an important challenge in the medical education of new physicians.

Many physicians cite their unfamiliarity with obesity as an impediment to their ability to adequately address the problem with their patients [3]. In one study by Jelalian et al., the authors found that when addressing obesity, one fourth of physicians thought that they were not at all or only slightly competent, while 20% reported feeling not at all or only slightly comfortable [3]. My impression is that obesity is not well represented in examinations of medical knowledge. As the latest confirmation of this hypothesis, I found it alarming that there were no questions addressing management of obesity on the American Board of Internal Medicine training examination in August 2004. This is my based on my immediate recollection of the exam, and confirmed by an informal same-day survey of five co-examinees. I later wrote to the ABIM to confirm my suspicion of this omission. In their response, they could not deny that the topic of obesity management had been left out [4]. I pose to you that our medical education is based largely upon that which we are tested. If this is true, it is not surprising that many physicians do not feel comfortable managing obesity.

I recalled at least five examination questions addressing Wegener's granulomatosis, but none directly on obesity. The differences in prevalence are remarkable. In the United States, approximately 61% (110 million) of adults (age 20–74 years) are overweight or obese. [5]. Educators must realize that if we are to aggressively arrest this epidemic, we must be armed with the knowledge to do so. Awareness, in part comes from exposure. By that, I mean, that which we study is that which we will be questioned on. If we all agree that obesity education is a priority, it follows that examinations should reflect that prioritization.

Diabetes, coronary heart disease, dyslipidemias, hypertension, and cancer were appropriately well represented in this ABIM exam and previous standardized examinations I have taken. Obesity should be right up there with these "big players". There is a lot to understand within this field. It is a lot more complex than "eat less, make better nutritional choices, and exercise more". The complexities of nutritional decision making are very important to the patient, and they want to discuss these choices with physicians as well as dieticians. Pharmacotherapy, although limited in terms of number of options, does have a role. Bariatric surgery is an evolving field and there are complexities associated with the use of the procedure that surgeons and internists alike must understand. Also, such psychosocial aspects of obesity as diminished quality-of-life, self-esteem, and work performance must be stressed and understood. Fogelman reported that in one survey of family practitioners, 72% believed that they had limited efficacy in treating obesity and considered themselves not well prepared by medical school to treat overweight patients [6]. Some 60% reported feeling that they have insufficient knowledge regarding nutritional issues. Regarding pharmacotherapy for treating obesity, only 66% knew the drugs' prescription indications. [6].

Primary prevention is the way to go in obesity research expenditure. In my opinion, the greatest healthcare cost-savings' "bang-for-the-buck" comes from preventive care and education. Consider how many fewer cases of diabetes, hypertension, CHD, fatty liver disease, dyslipidemia, thromboembolic disease, obesity-hypoventilation, obstructive sleep apnea, gout, osteoarthritis, cancer, GERD, reproductive and urinary tract abnormalities, cataracts, psychosocial abnormalities we would have to battle if the inciting factor (obesity) was aggressively treated at the onset. Is this not one prudent management step toward curtailing an overtaxed health care system?

The war on obesity seems winnable. We just need the tools and awareness to address the disease. Obesity is not a silent disease to be tolerated: it is an aggressive yet insidious disease (much like type 2 diabetes) that must be arrested before its negative health consequences become irreversible. The first step is to teach practitioners what to look out for, and in turn, to teach patients what to look out for. Meanwhile, I will have my antennas up in search of Wegener's granulomatosis.

## Competing interests

I hereby declare that I do not have a financial association or other conflict of interest with the subjects mentioned in this manuscript.
